# Production of Calcaride A by *Calcarisporium* sp. in Shaken Flasks and Stirred Bioreactors

**DOI:** 10.3390/md13073992

**Published:** 2015-06-24

**Authors:** Anu Tamminen, Yanming Wang, Marilyn G. Wiebe

**Affiliations:** VTT Technical Research Centre of Finland, P.O. Box 1000, FI-02044 VTT, Finland; E-Mails: anu.tamminen@vtt.fi (A.T.); yanming.wang@vtt.fi (Y.W.)

**Keywords:** marine fungi, *Calcarisporium*, calcaride A, stirred tank bioreactor, pH, macrocylic polyester

## Abstract

Increased interest in marine resources has led to increased screening of marine fungi for novel bioactive compounds and considerable effort is being invested in discovering these metabolites. For compound discovery, small-scale cultures are adequate, but agitated bioreactors are desirable for larger-scale production. *Calcarisporium* sp. KF525 has recently been described to produce calcaride A, a cyclic polyester with antibiotic activity, in agitated flasks. Here, we describe improvements in the production of calcaride A in both flasks (13-fold improvement) and stirred bioreactors (200-fold improvement). Production of calcaride A in bioreactors was initially substantially lower than in shaken flasks. The cultivation pH (reduced from 6.8 to <5.4), carbon source (sucrose replacing glucose), C/N ratio and nature of mycelial growth (pellets or filaments) were important in improving calcaride A production. Up to 4.5 mg·g^−1^ biomass (85 mg·L^−1^) calcaride A were produced in the bioreactor, which was only slightly less than in shaken flasks (14 mg·g^−1^, 100 mg·L^−1^). The results demonstrate that a scalable process for calcaride A production could be developed using an iterative approach with flasks and bioreactors.

## 1. Introduction

Organisms from marine environments, especially marine bacteria, actinomycetes, dinoflagelates, invertebrates and sponges, but also fungi, are recognized as good sources of novel, bioactive compounds [1,2,3]. Marine fungi produce a wide range of novel bioactive compounds and considerable effort is now being invested in discovering these metabolites [4]. Compound screening necessarily makes use of small-scale cultivation techniques, microtiter plates or flasks, with a limited range of environmental conditions. However, adequate supply is a problem for many marine derived compounds of clinical interest and methods for larger-scale cultivation and compound production are needed [2,4]. Scaling up the production of these products is often challenging because conditions used during screening are not suitable for larger-scale cultivations and often provide little information concerning the conditions needed to induce product formation or the time at which the product is formed. Obtaining adequate growth of the producing organism may also be challenging, particularly for filamentous organisms. However, recently, production of several diverse products from marine fungi has been successfully scaled up in stirred bioreactors (e.g., aspergiolide A [5], compound 1403C [6], scopularide A [7], and pigments [8]).

**Figure 1:**
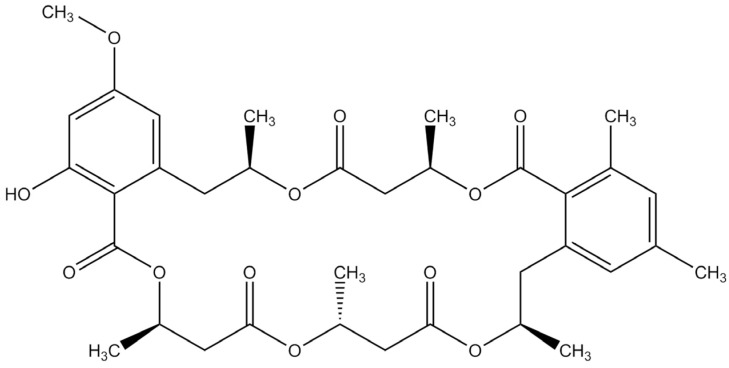
Chemical structure of calcaride A.

*Calcarisporium* species (e.g., *C. arbuscula*) are generally isolated as endophytes or parasites from agarics (Basidiomycetes) or higher Ascomycetes or from wood [9], but are occasionally isolated from marine environments [10]. Extracts from some species have been used as feed preservative [11] and bioactive compounds (e.g., macrocyclic trichothecenes, sesquiterpene esters, and sterols) have been identified from several isolates [12,13,14,15]. Recently, a strain (KF525) identified as *Calcarisporium* sp. was isolated from the German Wadden Sea and was shown to produce a range of macrocyclic and linear polyesters [10] and cyclic peptides [16]. Calcaride A (Figure 1), a macrocyclic polyester, was shown to inhibit growth of the plant pathogen *Xanthomonas campestris*, with some activity against *Staphylococcus epidermidis* [10]. Related compounds are reported to have antifungal activity [17], but the bioactivity of calcaride A has not been extensively investigated.

The growth characteristics of *Calcarisporium* species are poorly described. Hoog [9] described *Calcarisporium* Preuss as “growing moderately rapidly” on agar-solidified medium, but measurements of growth of other strains of *Calcarisporium* have not been reported. A *C. arbuscula* strain was reported to grow at 25 °C [14,15], producing metabolites within seven days, and *C. thermophilum* at 30 °C [13].

In this paper, we investigate the growth of *Calcarisporium* sp. KF525 and production of one of the five calcaride products, using calcaride A as an indicator of general calcaride production.

## 2. Results and Discussion

*Calcarisporium* sp. KF525 grew slowly in agitated liquid cultures at 22 °C, with a maximum specific growth rate of 0.03 ± 0.00·h^−1^ in defined medium with ammonium sulfate as the nitrogen source, 0.04 ± 0.00·h^−1^ in defined medium with glutamine supplied as the nitrogen source, and 0.06 ± 0.01·h^−1^ in complex media. Strain KF525 grew poorly at 24 °C, and did not grow at 25 °C, although it could survive. KF525 died when incubated at 26 °C. The relatively low temperature required for growth of KF525 would contribute to its low specific growth rate. The slow growth phenotype contributes to the difficulty of studying secondary metabolite production by KF525, since the growth phase is long.

KF525 was cultivated in casamino acid glucose medium (pH 6.8) with low agitation, as previously reported [10]. Calcaride A was extracted from the mycelium (1.1 mg·g^−1^, equivalent to 2.9 ± 0.2 mg·L^−1^), but essentially no calcaride A was found in the culture supernatant (0.04 ± 0.04 mg·L^−1^) after incubation for 21 days, even though Silber *et al.* [10] reported purification of 17.7 mg from 12 L culture supernatant (1.5 mg·L^−1^) after 24 days growth in the same medium. Biomass production in the flasks was low (5.2 ± 1.4 g·L^−1^ after 10 days of incubation), limiting the amount of calcaride A produced. Biomass production was improved by cultivating KF525 in a bioreactor (7.1 g·L^−1^ after 11 days) in the same medium (pH 6.8), but production of calcaride A was considerably reduced (0.02 mg·g^−1^ biomass, equivalent to 0.15 mg·L^−1^). No calcaride A was detected in the culture supernatant of the bioreactor culture and the related compounds were produced in proportionally small amounts, based on the UPLC chromatograms. We were thus interested to determine under which conditions calcaride A could be produced and whether it would be feasible to produce calcaride A in the bioreactor.

### 2.1. Calcaride A Production Is Affected by the Carbon Source

Casamino acids provide both carbon and nitrogen source. To assess whether the carbon source affected calcaride A production, KF525 was grown in defined medium with glucose, sucrose, fructose, maltose, lactose, malt extract or starch as the carbon source, with ammonium as the nitrogen source. KF525 grew on all carbon sources, producing the most biomass from starch (10.2 g·L^−1^) and the least from lactose (4.4 ± 0.1 g·L^−1^Table 1) after 25 days incubation.

Specific calcaride A production from glucose in defined medium (0.7 mg·g^−1^) was similar to that observed in casamino acid medium (1.1 mg·g^−1^), but the volumetric production (4.7 ± 0.4 mg·L^−1^) was higher because the biomass concentration (6.3 ± 0.4 mg·L^−1^) remained high in the culture (Table 1). Calcaride A production was slightly higher with sucrose (0.9 ± 0.1 mg·g^−1^, 7.2 ± 0.9 mg·L^−1^) or fructose (1.5 ± 0.1 mg·g^−1^, 8.1 ± 0.3 mg·L^−1^) as the carbon source than with glucose and was significantly (*p* < 0.05) reduced with other carbon sources (Table 1). A similar trend of slightly higher calcaride A production from sucrose than from glucose was also observed in casamino acid medium (data not shown) and sucrose was provided as the carbon source for further studies.

The calcarides are related to the 15G256 compounds from *Hypoxylon oceanicum*, another marine fungus, described by Schlingmann *et al.* [17], who suggested that the precursors were 6-hydroxymellein and β-hydroxybutyric acid, presumably via malonyl-CoA. 6-Methoxymellein would also be needed for the synthesis of the calcarides [10]. Mellein, hydroxymelleins and methoxymelleins are produced by a variety of fungi (e.g., [18]). Interestingly, sucrose has been shown to be a good substrate for production of mellein by the fungus *Aspergillus ochraceous*, although maltose and starch were found to be equally good or better substrates for producing 4-hydroxymellein [19]. The preference for synthesis of calcaride A on sucrose or fructose suggests that glucose may repress the synthetic pathway. Production was not enhanced on maltose or longer chains of glucose. Efficient hydrolysis of the maltose, dextin or starch, however, may also have resulted in glucose repression. The hydrolytic enzymes produced by *Calcarisporia* species have not been studied.

**Table 1:** Biomass and Calcaride A (specific and volumetric) produced by *Calcarisporium* sp. KF525 grown in defined medium for *ca.* 25 days in flasks (100 rpm, 22 °C) with various carbon sources.

Carbon Source	Biomass (g·L^−1^)	Calcaride A (mg·g^−1^)	Calcaride A (mg·L^−1^)
sucrose	7.7 ± 0.6 ^bcd^	0.9 ± 0.1 ^c^	7.2 ± 0.8 ^b^
fructose	5.4 ± 0.04 ^ab^	1.5 ± 0.1 ^d^	8.1 ± 0.3 ^b^
glucose	6.3 ± 0.4 ^abc^	0.7 ± 0.02 ^bc^	4.7 ± 0.4 ^ab^
maltose	5.1 ± 0.2 ^a^	0.05 ± 0.02 ^a^	0.3 ± 0.1 ^a^
malt extract	9.7 ^cd^	0.4 ^ab^	3.9 ^ab^
starch	10.2 ^d^	0.2 ^a^	1.9 ^a^
xylose	5.3 ^ab^	0.2 ^a^	0.9 ^a^
lactose	4.4 ± 0.1 ^a^	0.1 ± 0.03 ^a^	0.4 ± 0.1 ^a^

Calcaride A was extracted from the mycelia with ethyl acetate. Values are mean ± standard error of the mean (*n* = 2 or 9). Values in the same column with the same superscript (a to d) did not differ significantly (*p* > 0.05).

When grown in bioreactors for 21 days, with sucrose as the carbon source and yeast extract as the nitrogen source and a constant pH of 6.8 ± 0.2, KF525 produced more calcaride A from sucrose (0.02 mg·g^−1^ biomass, equivalent to 0.32 mg·L^−1^) than from glucose (0.01 mg·g^−1^ biomass, equivalent to 0.16 mg·L^−1^), but production was still very low and comparable to that previously observed in the bioreactor with casamino acids glucose medium. Biomass production was slightly higher on sucrose (13.4 ± 1.6 g·L^−1^) than on glucose (11.6 ± 1.2 g·L^−1^).

### 2.2. The Effect of pH on Calcaride A Production

**Figure 2:**
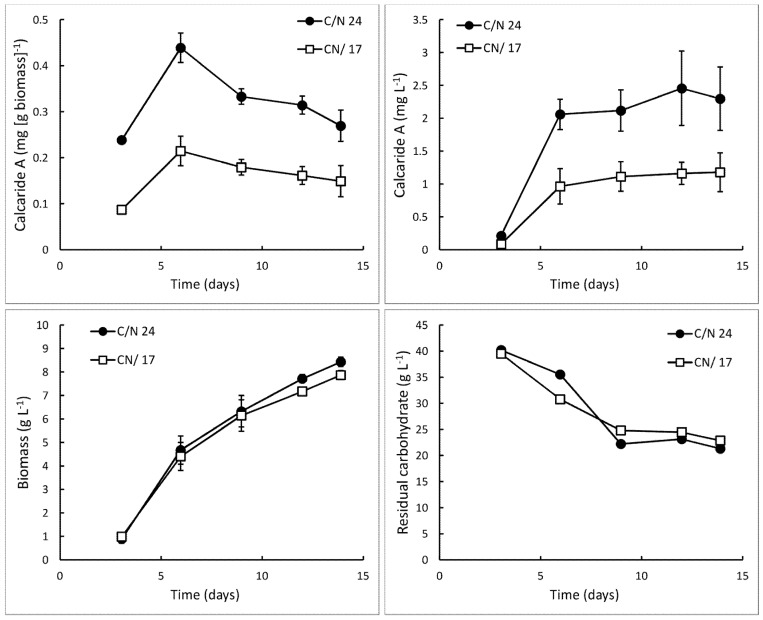
Production of calcaride A and biomass and total residual carbohydrate (*i.e*., glucose, fructose and sucrose) in bioreactor cultures of KF525 in defined medium with sucrose as the carbon source. The pH was maintained at 5.2 ± 0.3. Nitrogen was supplied as (NH_4_)_2_SO_4_ (C/N 24) or (NH_4_)_2_SO_4_ supplemented with glutamine (C/N 17). Error bars represent ± sem (*n* = 2).

**Figure 3:**
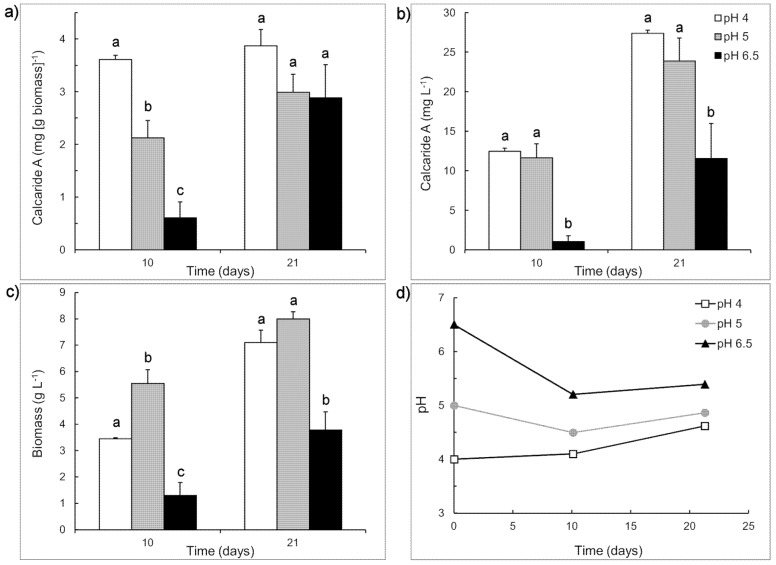
Production of (**a**) specific and (**b**) volumetric calcaride A and (**c**) biomass by KF525 in sucrose-casamino acid medium with initial pH of 4 (white), 5 (grey) or 6.5 (black). (**d**) Changes in pH during the cultivation. Error bars represent + sem (*n* = 2 or 4) and are smaller than the size of the symbol for pH. Letters above the bars (a, b or c) indicate significant differences (*p* < 0.05) for values measured at the same time (Day 10 or Day 21).

Since production in defined medium in agitated flasks was as high as in complex (casamino acid glucose) medium in flasks, *Calcarisporium* sp. KF525 was also grown in defined medium in the bioreactor. The pH of defined medium is lower than that of the casamino acid glucose medium described by Silber *et al.* [10], and these cultures were grown at pH 5.2 ± 0.3, the initial pH of the medium. Sucrose was provided as the carbon source. Within only 14 days, 0.3 ± 0.0 mg·g^−1^ (equivalent to 2.3 ± 0.5 mg·L^−1^; Figure 2) calcaride A was produced, which was approximately ten times higher than previously obtained in a bioreactor. However, the carbohydrate was not completely consumed (9.0 ± 0.5 g·L^−1^ glucose and 13.0 ± 0.8 g·L^−1^ fructose remaining after 12–14 days), and only 8.4 ± 0.7 g·L^−1^ biomass was produced (Figure 3, C/N 24).

To assess the importance of the shift from pH 6.8 to pH 5.2 for obtaining calcaride A production in the bioreactor, we compared the production of calcaride A at different initial pH values in flasks using the casamino acid medium described by Silber *et al.* [10] and adjusting the initial pH to either 6.8 (pH 6.5 after sterilization) or 5.2 (pH 5.0 after sterilization). The comparison was made with sucrose as the carbon source and a lower pH (4.0) was included. During growth, the pH of cultures with initial pH of 6.5 decreased to 5.4 ± 0.0, whereas the pH of cultures with initial pH of 4 increased to 4.6 ± 0.1 and the pH of cultures with initial pH of 5 changed very little (Figure 3).

Production of calcaride A (volumetric) was similar at pH 4 and 5 and significantly (*p* < 0.05) lower at pH 6.5 after 10 and 21 days incubation, reflecting both higher initial (*i.e*., at day 10) specific concentrations of calcaride A in the mycelia at pH 4 or 5, compared to pH 6.5, and higher biomass production (Figure 3). The amount of calcaride A produced in casamino acid sucrose medium (Figure 3) was higher than that previously observed in defined medium with sucrose as the carbon source (Table 1), with up to 3.9 ± 0.3 mg·g^−1^ biomass (equivalent to 27.4 ± 0.4 mg·L^−1^) calcaride A produced at pH 4 after 21 days incubation. Glucose and/or fructose, but no sucrose, were still present after 21 days of incubation in cultures with initial pH 4 (3.9 ± 0.0 g·L^−1^ glucose), pH 5 (6.5 ± 0.1 g·L^−1^ fructose) and pH 6.5 (9.7 ± 0.9 g·L^−1^ glucose, 16.2 ± 0.4 g·L^−1^ fructose). Since the pH of cultures with initial pH 4 increased during incubation, and volumetric calcaride A production was similar at pH 4 and 5, pH values around 5 were used for subsequent investigations.

Both calcaride A and biomass were preferentially produced at low pH, explaining why production in defined medium, with initial pH ~5.2, was generally better than in medium with organic nitrogen sources (casamino acids or yeast extract), with initial pH 6.5 to 7. It also explained why production in complex medium in flasks with initial pH 6.5–6.8 was better than in bioreactors at pH 6.8 (Section 2.1), since the pH in the flasks decreased to levels at which calcaride A production would be enhanced, whereas the pH of the bioreactors had been kept constant. While many filamentous fungi prefer to grow at pH values between 4 and 6, marine fungi are often grown in media with initial pH values between 6 and 7 when screening for compounds, presumably because the marine environment would be slightly alkaline (pH 7.5–8.4). Some compounds are optimally produced in this range (e.g., [6,8]), but the optimum is likely to be strain and product specific.

### 2.3. The Effect of Nitrogen and Carbon Concentrations on Calcaride A Production

The casamino acid glucose medium described in [10] does not supply sufficient nitrogen for all of the carbon to be consumed unless carbon is incorporated into storage compounds or secondary metabolites. The carbon–nitrogen ratio (C/N) of this medium was ~67 (g/g), which was much higher than the level at which nitrogen is expected to limit growth (C/N > 10; [7,20]). A high C/N ratio may be useful to trigger production of secondary metabolites such as the calcarides that do not contain nitrogen, and we continued to use medium with high C/N ratios. However, sucrose was not completely consumed in complex (C/N ~67, Section 2.2) or defined (C/N ~24, Figure 2) medium, suggesting that the C/N ratio could be reduced, either by increasing the amount of nitrogen supplied or by reducing the carbon concentration. When the concentration of nitrogen in the defined medium was increased (C/N ~17), supplied as either more ammonium or as the amino acid glutamine, sucrose consumption and biomass production were essentially unaffected, indicating that nitrogen remained limiting, while calcaride A production was reduced by about 50% (Figure 2). We therefore considered C/N ratios between ~24 and ~67.

**Figure 4:**
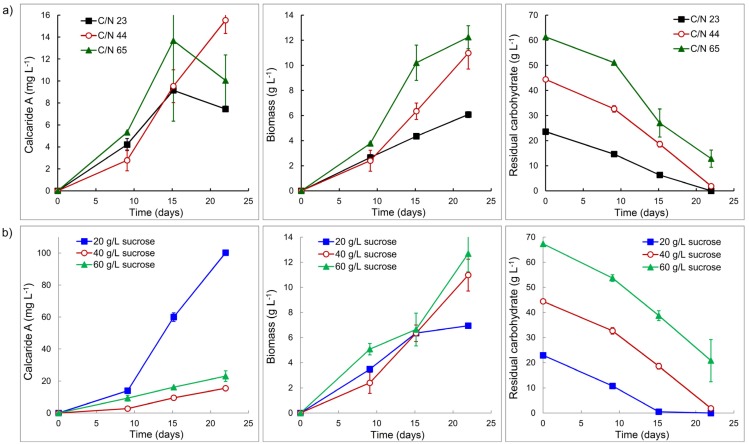
(**a**) Production of calcaride A and biomass by KF525 and residual carbohydrate (sucrose, glucose and fructose) concentration in sucrose glutamine (2 g·L^−1^) medium with initial pH of 5 and variable C/N. (**b**) Production of calcaride A and biomass by KF525 and residual carbohydrate concentration in sucrose glutamine medium with initial pH of 5 and C/N ratio of 44. Error bars represent ± sem (*n* = 2).

Two sets of conditions were investigated in flasks, either keeping the concentration of the nitrogen source (glutamine) constant but changing the concentration of sucrose to change the C/N ratio or adjusting the concentration of glutamine to maintain a constant C/N ratio for different concentrations of sucrose. Glutamine was used as the nitrogen source since results obtained while reviewing the protocol to generate conidia of KF525 indicated that glutamine supported better production of calcaride A than ammonium or casamino acids. Amino acids have also been shown to affect production of mullein and 4-hydroxymellein in *A. ochraceous* [19]. Production of calcaride A in flasks with a constant concentration of glutamine, but different concentrations of sucrose (20, 40 or 60 g·L^−1^ to provide C/N 23, 44 or 65), was not affected (*p* > 0.05) by the change in C/N (Figure 4a). The yield of biomass from sucrose (0.26 g biomass per g sucrose consumed) was the same at all C/N ratios, but significantly (*p* < 0.05) less biomass was produced from 20 g·L^−1^ sucrose (C/N 23) than from 40 or 60 g·L^−1^ sucrose. Carbohydrate (sucrose, glucose or fructose) was not completely consumed at C/N 65 with 60 g·L^−1^ sucrose provided, but was consumed at C/N 44 and 23 (Figure 4a). Since all carbohydrate was consumed at C/N 44, we used this ratio of carbon and nitrogen to assess whether providing more or less sucrose could be beneficial.

Reducing the C/N from 65 to 44 with 60 g·L^−1^ sucrose did not enable KF525 to utilize all the carbohydrate (Figure 4b) and production of calcaride A and biomass was similar (*p* > 0.05) at C/N 44 and C/N 65 (Figure 4). However, increasing the C/N from 23 to 44 with 20 g·L^−1^ sucrose resulted in a significant (*p* < 0.05) increase in calcaride A production (Figure 4b), although biomass production was not (*p* > 0.05) affected. All carbohydrate was consumed within 15 days. High calcaride A production thus occurred during a carbon-limited stationary phase, as might be expected for a secondary metabolite. Calcaride A production was significantly higher (*p* < 0.05, ANOVA) than in flasks with 40 or 60 g·L^−1^ sucrose after the sucrose had been consumed (Figure 4b). Biomass production after 21 days with 40 g·L^−1^ sucrose and 2 g·L^−1^ glutamine (C/N 44, Figure 4) was comparable (*p* > 0.05) to that obtained with 40 g·L^−1^ sucrose and casamino acids at pH 4–5 (C/N ~67, Figure 3), but calcaride A production was lower (*p* < 0.05).

### 2.4. Improved Production of Calcaride A in Bioreactor Cultures

**Figure 5:**
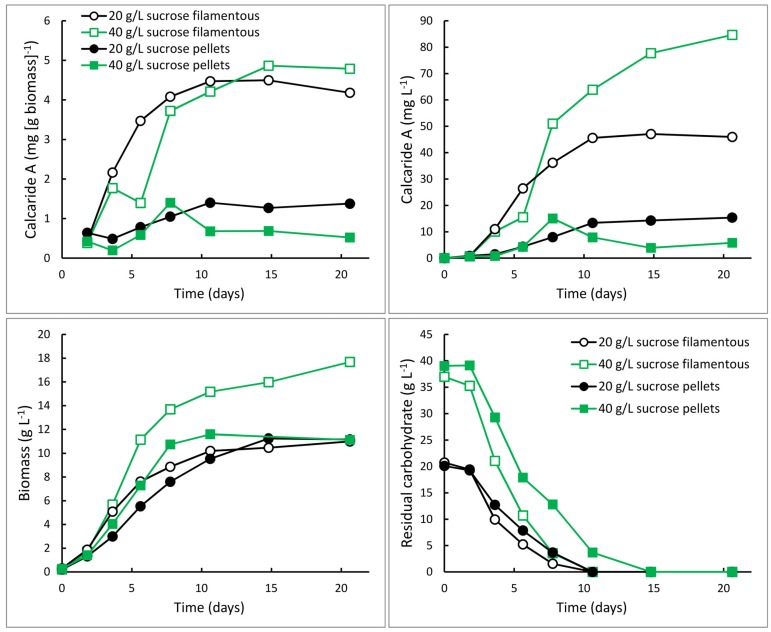
Production of calcaride A and biomass and consumption of carbohydrate (sucrose and glucose and fructose derived from sucrose) by KF525 in bioreactors with 20 (circles) or 40 (squares) g·L^−1^ sucrose, and nitrogen supplied as glutamine (C/N 44, pH 5.4 ± 0.1). The mycelium grew as pellets (solid symbols) or filamentously (open symbols).

Reducing the pH from 7.0 to 5.2 had resulted in measureable calcaride A production in stirred tank reactors (Figure 2), but production was lower than observed in the equivalent flasks. The best production in flasks was obtained in medium with 20 g·L^−1^ sucrose and glutamine as the nitrogen source with a C/N ratio of 44. When this condition was applied in a bioreactor (pH 5.4), calcaride A production increased to 1.4 mg·g^−1^ (16.2 mg·L^−1^; Figure 5, 20 g/L sucrose pellets). This was comparable to the production observed after 21 days in flasks with 40 or 60 g·L^−1^ sucrose (Figure 4). Calcaride A was produced during growth, and no further calcaride A production occurred after the carbohydrate had been consumed (11 days) (Figure 5). Although production was lower than in flasks, the time required to produce calcaride A was reduced by nearly half. Production of calcaride A was similar at pH 4 as at pH 5.4 (data not shown). When the concentration of sucrose was increased to 40 g·L^−1^, calcaride A production was similar to that observed with 20 g·L^−1^ sucrose during the first eight days, but then decreased (Figure 5, 40 g/L pellets).

KF525 generally grew as pellets in flasks and inclusion of agar to obtain filamentous growth (*cf.* [7]) appeared to suppress calcaride A production (data not shown). Therefore, bioreactors were inoculated with pre-cultures containing small pellets and the cultures continued to grow as small pellets (1–3 mm diameter). Unexpectedly, pre-cultures for two sucrose glutamine cultivations were filamentous and grew filamentously in the bioreactors. These cultivations grew more rapidly than the cultures that consisted of pellets and they consumed the substrate efficiently (Figure 5), as expected for filamentous compared to pellet growth. Unexpectedly, they also produced more calcaride A (~4.5 mg·g^−1^) than cultures with pellets (Figure 5). All sucrose was consumed, even when 40 g·L^−1^ sucrose was supplied, resulting in a higher biomass concentration and consequently higher volumetric production of calcaride A with 40 compared with 20 g·L^−1^ sucrose. The amount of calcaride A produced in the filamentous bioreactor culture with 40 g·L^−1^ sucrose (85 mg·L^−1^) was comparable to that obtained from flasks with 20 g·L^−1^ sucrose and 1 g·L^−1^ glutamine (100 mg·L^−1^). Neither bioreactor cultures with pellets nor bioreactor cultures with filamentous mycelia were oxygen limited, but nutrient (sucrose or oxygen) diffusion through the pellets may be limiting calcaride A production in stirred tank reactors. It was not clear why calcaride A was produced well by pellets in flasks, even when shaken at only 100 rpm, but not in bioreactors. Furthermore, additional study would be needed to develop a protocol to consistently generate inocula with the correct morphology for bioreactor production of calcaride A. None-the-less, the results demonstrate that production of substantial amounts of calcaride A in bioreactors is feasible.

A variety of strategies have been applied to obtain production of secondary metabolites from marine fungi in stirred tank bioreactors. Aspergiolide A production required a low shear environment, adequate oxygen [5] and pH near 7 [21]. Zhou *et al.* [6] and Tamminen *et al.* [7] found that conditions which promoted good biomass production promoted the production of 1403C (an anthracycline analogue) and scopularide A, respectively. The carbon–nitrogen ratio is important in the regulation of both fatty acid and polyketide synthesis and was optimized to enhance production of oxalicumone A [22]. Both carbon and nitrogen sources may be important [8,22].

The C/N ratio and the source of carbon were both important in production of calcaride A. The nitrogen source may also be important, but was not systematically investigated. Because of the slow growth of *Calcarisporium* sp. KF525 and the need to maintain a relatively high C/N ratio, it was not possible to improve calcaride A production by increasing biomass production by KF525 or by using fed-batch strategies. The observation that filamentous mycelia were more productive than pellets suggests that agitation and the state of the pre-cultures are important and should be investigated further. Indeed it should be possible to further enhance calcaride A production in bioreactors now that detectable production has been achieved.

## 3. Experimental Section

### 3.1. Strain

*Calcarisporium* KF525 was obtained as a kind gift from A. Labes, from the culture collection of the Kiel Center for marine natural products at GEOMAR, Helmholtz Centre for Ocean Research Kiel. Stock cultures were maintained as conidia suspended in 20% v/v glycerol, 0.8% w/v NaCl with ~0.025% v/v Tween 20 at −80 °C. Conidia were obtained from cultures grown in shaken flasks on Yeast Malt Peptone (YMP, [23]) medium containing 30 g·L^−1^ Sea Salt (Tropic Marin^®^, Germany) for 11 days, after removal of the mycelia by filtration through cotton. Conidia were collected by centrifugation, resuspended in the glycerol-NaCl-Tween20 solution, and stored frozen at −80 °C.

### 3.2. Media

*Calcarisporium* KF525 was grown in casamino acid glucose (2.5 g·L^−1^ casamino acids, 40 g·L^−1^ glucose, 1.8 g·L^−1^ KH_2_PO_4_, 0.1 g·L^−1^ MgSO_4_·7H_2_O [10]), YMP (3 g·L^−1^ yeast extract, 3 g·L^−1^ malt extract and 5 g·L^−1^ soy peptone, [23]), modified Vogel’s ([24], with (NH_4_)_2_SO_4_ substituted for NH_4_NO_3_) or Yeast Nitrogen Base (YNB without amino acid and without ammonium sulfate, Becton, Dickinson and Company, Franklin Lakes, USA) media with glutamine as the nitrogen source. Glucose, fructose, sucrose, maltose, malt extract, starch, xylose, or lactose (40 g·L^−1^, unless otherwise stated) were provided as primary carbon sources. Agar solidified media contained 15 g·L^−1^ agar. Some media for pre-cultures also contained 4 g·L^−1^ agar to facilitate filamentous growth, but this was found to delay calcaride A production in the production medium. All media contained 30 g·L^−1^ Tropic Marin^®^ Sea Salt.

For determination of approximate C/N ratios, yeast extract was assumed to contain 9.8% total N and casamino acids to contain 10% total N [25]. Peptides (amino acids) were assumed to contain 47% carbon.

### 3.3. Cultural Conditions

Flasks (250 or 500 mL, containing 50 or 100 mL medium) were inoculated with conidial suspensions to give final concentrations of ~2 × 10^5^ conidia mL^−1^ and incubated at 22 °C, 120–200 rpm, for up to 25 days. Pre-cultures for bioreactors were incubated at 200 rpm for 3 to 5 days. Bioreactors were inoculated with 10% final volume.

*Calcarisporium* KF525 was grown in 1 L (Biostat Qplus, max working volume 1.0 L, Sartorius AG, Göttingen, Germany) and 2 L (Sartorius Biostat B or CT2, max working volumes 2 L, Germany) bioreactors. Bioreactors were maintained at 22 °C, with 200 to 500 rpm agitation and 0.4–1.5 volume gas (volume culture)^−1^·min^−1^ (vvm). Culture pH was kept constant by the addition of sterile 1 M NaOH or 1 M H_3_PO_4_. Polypropylene glycol (1:1 mixture of M_n_ ~1000 and M_n_ ~2000, [26]) was added to control foam formation. When measured, gas concentration (CO_2_ and O_2_) was analyzed continuously using a Prima Pro Process mass spectrometer (Thermo Scientific, Winsford, UK) calibrated with 3% CO_2_ in Ar, 5% CO_2_ with 0.99% Ar and 15% O_2_ in N_2_, 20% O_2_ plus 20% Ar in N_2_, and 0.04% ethanol in N_2_.

Samples were removed at intervals and mycelium was separated from the supernatant by centrifugation (3500 rpm, 15 min, Eppendorf AG, centrifuge 5810 R, Hamburg, Germany). Mycelia were washed twice in water by centrifugation and freeze-dried (Christ, Freeze Drier, alpha 1-4 LD plus, Osterode am Harz, Germany) to determine the biomass dry weight. Freeze-dried biomass was used for compound extraction.

### 3.4. Chemical Analyses

Calcarides were extracted from mycelia and/or culture supernatant with ethyl acetate. Freeze-dried mycelia (10–50 mg) were fragmented in the presence of 1.8 mL ethyl acetate and 2 steel balls in a MM301 ball mill (2 × 3 min at 20 1/S, Retsch GmbH, Haan, Germany). Samples were centrifuged for 10 min at 10,000 rpm (Eppendorf AG centrifuge 5430) and the ethyl acetate phase transferred to a clean microfuge tube. Alternatively, calcarides were extracted from 20 mL culture supernatant, from which mycelia had been removed by centrifugation, by vortexing with 20 mL ethyl acetate. Phases were separated by centrifugation (3500 rpm, 15 min, Eppendorf AG, centrifuge 5810 R) and the ethyl acetate layer was retained. Ethyl acetate was evaporated under nitrogen and the solids re-dissolved in 200 µL HPLC grade methanol.

Ethyl acetate extracts were analyzed by UPLC (ACQUITY UPLC, Waters) using a C18 UPLC column (1.7 µm, 2.1 mm × 100 mm, Waters; solvent isocratic (A) 70% (v/v) acetonitrile, (B) 5% (v/v) formic acid in H_2_O), and detected with a UV detector. Calcaride A was quantified using a pure sample kindly provided by Antje Labes (GEOMAR, Kiel, Germany).

The concentration of glucose, fructose, or sucrose in culture supernatant was determined by HPLC using a Fast Acid Analysis Column (100 mm × 7.8 mm, BioRad Laboratories, Hercules, CA, USA) linked to an Aminex HPX-87H organic acid analysis column (300 mm × 7.8 mm, 35 °C, BioRad Laboratories) with 5 mM H_2_SO_4_ as eluent and a flow rate of 0.5 mL·min^−1^. Peaks were detected using a Waters 410 differential refractometer.

### 3.5. Statistical Analyses

Data are presented as mean ± standard error of the mean when replicates were available. Replicates were not included for flask cultivations with low production of calcaride A, nor was it possible to replicate all bioreactor cultivations because of the time required for each experiment. When only two conditions were being compared, the student t test was applied. For comparison of three or more means, Analysis of Variance was applied and significant differences were identified using Fisher’s multiple range test. Significance levels are indicated in the text.

## 4. Conclusions

Calcaride production has been improved in flasks and transferred to a stirred tank bioreactor environment. Initially, no production of calcaride A was obtained in bioreactors, but comparison with the flask cultures demonstrated that calcaride A production was sensitive to pH, carbon source and the carbon–nitrogen ratio. Calcaride A was produced best at pH values below 5.4 with sucrose as the carbon source. Complex medium was not required. Production of calcaride A in flasks was increased 13-fold per gram biomass, with a corresponding 34-fold improvement in volumetric production. The best production obtained in a bioreactor (4.5 mg·g^−1^, 85 mg·L^−1^) was substantially (200–300 times) higher than that initially observed and only slightly lower than that obtained in the best conditions in flasks (14 mg·g^−1^, 100 mg·L^−1^). During screening, investigators typically focus only on one (usually glucose) or a few carbon or nitrogen sources. The results presented here demonstrate that these conditions may limit the products obtained. However, too little is yet known to develop physiologically rationalized screening approaches to stimulate production of the desired classes of compounds.
